# Reasons for Declining Consent in a Population-Based Cohort Study Conducted in a Rural South American Community

**DOI:** 10.1155/2018/8267948

**Published:** 2018-11-26

**Authors:** Oscar H. Del Brutto, Pablo R. Castillo, Mark J. Sedler, Victor J. Del Brutto, Mauricio Zambrano, Robertino M. Mera, Clinton B. Wright, Tatjana Rundek

**Affiliations:** ^1^School of Medicine, Universidad Espíritu Santo and Ecuador, Guayaquil, Ecuador; ^2^Sleep Disorders Center, Mayo Clinic School of Medicine, Jacksonville, FL, USA; ^3^School of Medicine, Stony Brook University, New York, NY, USA; ^4^Department of Neurology, University of Chicago, Chicago, IL, USA; ^5^Community Center, The Atahualpa Project, Atahualpa, Ecuador; ^6^Vanderbilt University Medical Center, Nashville, TN, USA; ^7^National Institute of Neurological Disorders and Stroke, Bethesda, MD, USA; ^8^Department of Neurology, Miller School of Medicine, University of Miami, Miami, Fl, USA

## Abstract

There is limited information on participants' adherence and reasons for declining consent in observational cohort studies conducted in remote rural communities. We aimed at sharing lessons learned during the Atahualpa Project, a population-based cohort study conducted in a rural Ecuadorian village. Atahualpa residents aged ≥40 years identified during door-to-door surveys who signed a consent form were enrolled. Annual surveys were conducted to assess the number of participants who moved out of the village, as well as those who died, declined consent, and newly entered the study. Reasons for declining consent were tabulated. Abstracted data included age, sex, education, disability, time between enrollment and declining consent, and reasons for withdrawal. We also counted participants who, despite expressing their willingness to continue in the study, refused specific procedures. After five years of follow-up, 54 (6.3%) of 863 enrolled individuals declined consent. Increasing age and disability had no impact on declining consent. In contrast, refusal was higher among relatives or neighbors of a given participant declining consent. Most people who declined consent did so after one or two years of enrollment. Less than 20% of enrolled individuals refused certain procedures. “Fear of the needle” was the most frequent reason for refusing blood tests, and common reasons for declining complimentary exams were lack of interest and time constraints. Cohort retention in the Atahualpa Project is high. Main reasons for this adherence include adequate selection of the village, detailed planning of procedures, assurance of sponsorship, and field personnel who continuously engage with study participants. This trial is registered with NCT01627600.

## 1. Introduction

Enrollment of participants in population-based research studies is the most basic element of a successful project. Maintaining enrolled individuals in the cohort is equally essential for the successful observation of longitudinal exposures and outcomes. Retention of study individuals can be very challenging in long-term studies, even when such studies provide medications or other interventions [1–4]. This challenge is even greater in observational cohort studies where participants are subject to repetitive interviews and time-consuming diagnostic tests during the follow-up, when no intervention other than advice or counseling is offered [5–7].

Conducting an observational cohort study in remote rural settings of low- and middle-income countries (LMIC) is particularly challenging due to illiteracy, cross-cultural factors, limited availability of sophisticated diagnostic tools, and lack of funding. Moreover, there is limited information on the burden and long-term public health impact of noninfectious cardiovascular and neurological diseases in rural areas of Latin America [8–10], and certainly, there are no population-based cohort studies addressing participants' adherence and the reasons for declining consent in these regions. Using the Atahualpa Project cohort, we aimed at describing the percentage and the reasons for declining consent of participants from this population-based cohort study conducted in a rural Ecuadorian village. The following commentaries also aim at providing some useful insights for investigators who plan to embark on global health research by sharing lessons learned from the conduct of the present study.

## 2. Materials and Methods

Atahualpa is a rural village located in coastal Ecuador that was selected as representative of the villages of the region. The Atahualpa population is homogeneous regarding race/ethnicity, living conditions, socioeconomic status, and dietary habits, as detailed elsewhere [11]. Most men work as artisan carpenters in the village, and almost all women are homemakers. The village has a low migration rate, which makes it an optimal setting for conducting longitudinal population-based cohort studies [12].

In June 2012, we started the Atahualpa Project, a population-based cohort study that was mainly designed to assess prevalence, incidence, and correlates of major neurological and cardiovascular disorders in community-dwellers aged ≥40 years. Before the start of the study, the Executive Board of the Atahualpa Project met village authorities and community leaders to explain the aims of the study and to ensure the people's interest in participation and complying with follow-up assessments. Then, field personnel conducted a baseline door-to-door survey to identify Atahualpa residents aged ≥40 years, defined as persons who had lived in the village for at least three months preceding the start of the survey. Field personnel were carefully selected from villagers based on their understanding of the population and were trained during two weeks by our senior investigators in the use of instruments to assure uniformity of data. After a comprehensive informed consent process, eligible individuals who accepted and signed a written consent form were enrolled. The study was approved by the Institutional Review Board (IRB) of Hospital-Clínica Kennedy, Guayaquil (FWA 00006867).

Soon after the start of the study, we built a community center in the village that is staffed with medical and paramedical personnel. Enrolled individuals voluntarily attended the center to get medical check-ups and counseling on their symptoms and conditions. Every other year, participants aged ≥60 years have been invited to the center to receive advice on the control of cardiovascular risk factors. Our nutritionists and medical students present 30-minute counseling sessions weekly to the villagers; about 20 members of the community aged ≥60 years were invited every week during five consecutive months. Same participants are invited to attend the three different programs, with different topics, i.e., recommendations for a healthy diet, the importance of physical activity, and how to prevent complications of diabetes. No specific interventions—other than information on how to improve health behaviors and to reduce relevant risk factors—have been provided.

Throughout the study, Atahualpa residents have been periodically visited at their homes to record information about disorders of interest, allowing our personnel to maintain frequent contact with villagers. In addition, participants have been invited for complimentary exams (blood tests, sonographic examinations, ECGs, polysomnography, and direct fundoscopic examinations) to better assess the burden of diseases of interest. These exams were performed at the field research center. For participants needing help because of disabilities (mainly stroke patients or very old participants), field personnel assisted with the transportation from their homes to our center.

From the second year of the study, we implemented the Atahualpa Project Neuroimaging Substudy, which consisted of a nonenhanced head CT offered to all participants and a brain MRI and MRA of intracranial vessels to the subset of participants aged ≥60 years as well as to younger individuals presenting with an overt stroke, seizures, neurocysticercosis, or other neurological disorders of interest (and age-matched healthy controls in selected cases). For these assessments, participants are transported to Guayaquil (a city located about 100 miles away from Atahualpa). Individuals were provided with free transportation and meals, but no monetary compensation.

Annual door-to-door surveys have been conducted to assess the number of participants who moved out of the village, as well as the number of people who died, who declined consent, and those who newly entered the study (either because they moved into the village, reached an age appropriate to be enrolled, or volunteered after an initial refusal). In addition, the incidence of disorders of interest was assessed during these surveys [13].

Reasons for declining consent were tabulated. Abstracted data on these people included age, sex, level of education, presence of an overt stroke or disability, time between enrollment and declining consent, and the reason for withdrawal. For calculating the follow-up of individuals who declined consent after baseline, we considered the time under surveillance starting from the day of the first visit and ending at the administrative date of the last completed survey. In addition, we counted the participants who, despite expressing their willingness to continue in the study, refused specific complimentary exams. Results of this study included data from the baseline door-to-door survey as well as subsequent surveys conducted over five years (2013 to 2017).

## 3. Results

Of 666 Atahualpa residents aged ≥40 years identified during the baseline door-to-door survey, only 24 (3.6%) refused to participate. The main reason for refusal was lack of interest in 18 cases, and the remaining six reported time constraints. The initial phase of the study consisted of face-to-face interviews for the assessment of demographics and cardiovascular risk factors, as well as determinations of fasting glucose and total cholesterol blood levels from a capillary blood sample. None of the 642 participants initially enrolled refused these tests.

### 3.1. Definitive Refusals

**Figure 1:**
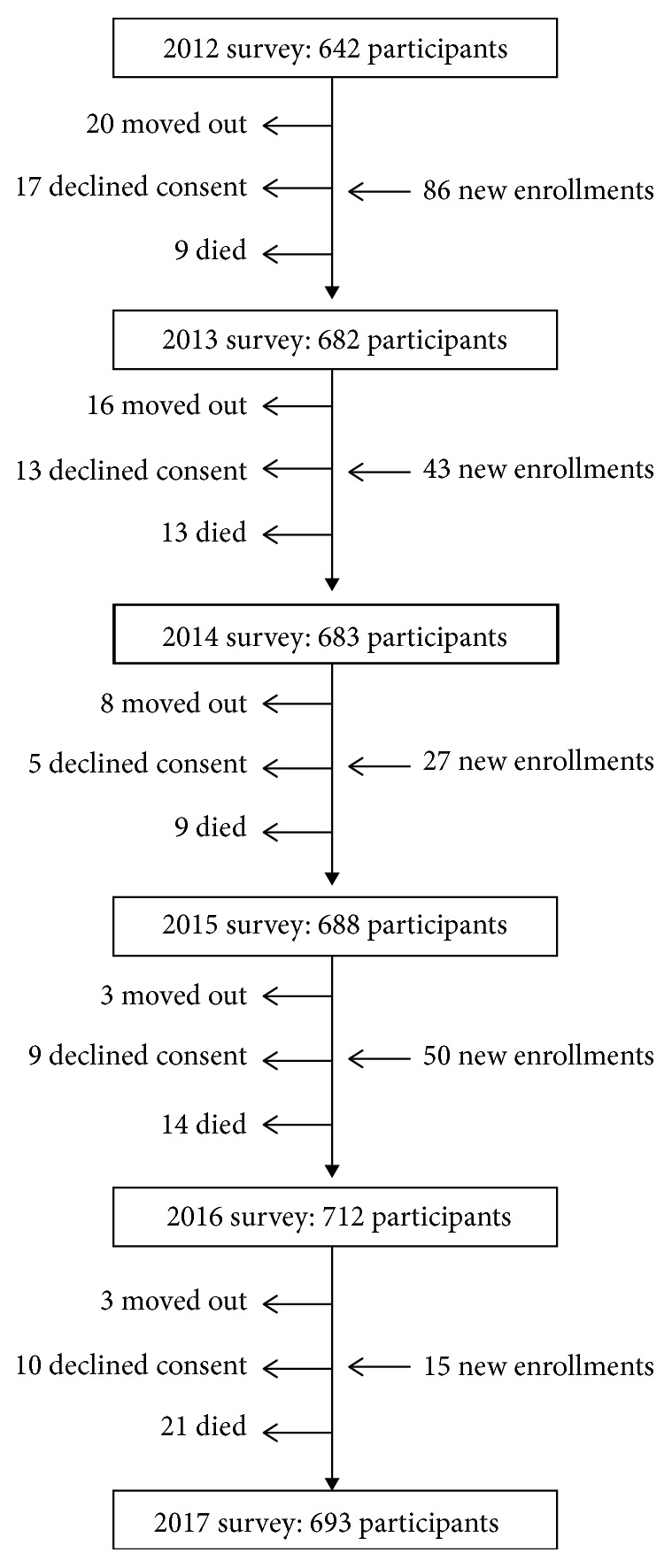
Algorithm showing dynamics of enrollment and missing participants in the Atahualpa Project during the study years (2013–2017).

Figure 1 shows dynamics of enrollment and the number of new and missing participants over the study years. A total of 54 (6.3%) out of 863 enrolled individuals declined consent and definitively refused to continue in the cohort. The mean age of these 54 individuals (at the time of enrollment) was 62.9 ± 12.2 years, 26 (48.1%) were women, and 34 (63%) had primary school education. In addition, only one (1.9%) out of 49 participants with an overt stroke or severely disabled declined consent. Characteristics of persons who refused were not significantly different from those who continued in the cohort (Table 1). The mean follow-up of the 54 individuals who withdrew consent was 2.6 ± 1.4 years, with most of them (62.5%) declining consent after one or two years of enrollment.

**Figure 2:**
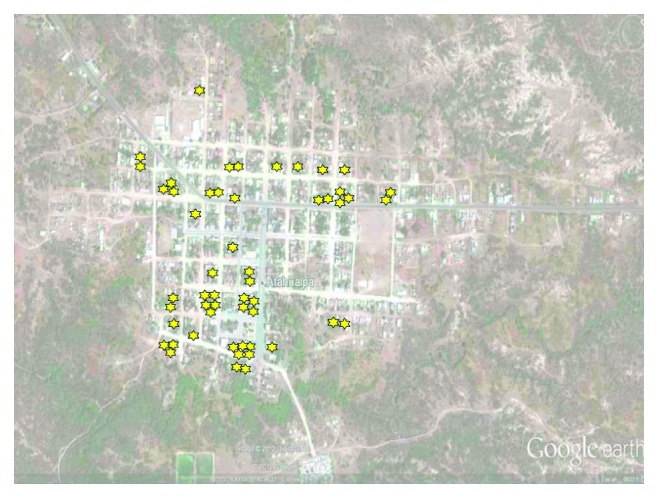
Satellite view of Atahualpa (Google Earth, Google Inc., Mountain View, CA), showing location of participants who declined the consent (stars).

The most frequent reason for declining consent was lack of interest in the study (30 cases), followed by the fact that free medications were not provided (13 cases) and perceived time constraints (10 cases); the remaining person was a woman whose husband prohibited her to continue in the study. Permanent refusal was recorded in 27 (28%) out of the 96 identified village blocks (Figure 2) and in only 41 (4%) out of the 1,009 inhabited houses. In 16 of these houses, more than one participant declined consent (most often husband and wife) at the same time.

### 3.2. Partial Refusals

During the study years, several interviews and procedures have been performed according to the protocol of the Atahualpa Project and, as previously mentioned, some individuals refused certain tests (but not all) despite their interest in continuing in the study. While this was not considered a total refusal and, in fact, these individuals were not excluded from the study, it is important to better understand which exams were more (or less) accepted by participants and the reasons for refusal. Those interviews and procedures can be grossly classified into the following groups: (1) face-to-face interviews using field instruments, (2) blood tests, (3) neuroimaging studies, (4) sonographic studies, (5) neurophysiological studies, (6) cardiac physiological tests, and (7) community talks for prevention of risk factors.  Table 2 is a summary of procedures and diagnostic tests performed during the follow-up, the percentage of acceptance based on the number of invited participants, and the main reasons for declining consent.

#### 3.2.1. Face-to-Face Interviews

Over the study years, several interviews were performed in participants for different reasons. Results of these interviews, as well as the field instruments used in each case, have been published elsewhere [14–18]. Generally, acceptance of these interviews has been high as they almost always were done at the home of participants (and field personnel returned two or three times if a given subject was not at home at the time of the first visit). As previously mentioned, baseline interviews on demographics and cardiovascular health status were offered to all the 863 participants at the time of enrollment and were collected from all participants. Six months after the baseline survey, an oral examination and interviews to assess sleep-related symptoms were offered to all participants. Going forward, these tests have been incorporated into the baseline interviews for new enrollees. As a result, with the exception of 36 participants who declined consent, moved out of the village, or died during the first six months of the study, all the remaining 827 individuals accepted the oral exam and sleep interviews (100% acceptance). Other field instruments that have been administered to the entire cohort since the 2013 survey, including the assessment of oily fish intake, measures of psychological distress, and seizure occurrence, and have been completed by approximately 95% of eligible participants. Even more complex interviews, such as the assessment of cognitive performance (using the Montreal Cognitive Assessment), were answered by 756 (96%) out of 787 eligible participants (excluding aphasic, deaf, and blind people).

#### 3.2.2. Blood Tests

With the exception of the first invitation to obtain a blood sample (conducted in 2012), where all stroke-free individuals (*n* = 616) were invited and 517 (84%) consented [19], these exams had not been offered to the entire population but only to people aged ≥60 years. Blood tests were performed in different years during the study, so the number of eligible candidates varied according to changes in the population dynamics. Three subsequent studies requiring blood tests were performed in the subset of people aged ≥60 years, and the percentages of acceptance was 82% (220/267) for 2014, 74% (259/351) for 2015, and 90% (319/351) for 2016 [20–22]. Major reasons for refusal included fear of the needle, although others reported being too busy to go to our center during working hours (blood tests were not performed at participants' homes).

#### 3.2.3. Neuroimaging Studies

A total of 701 CTs and 446 MRIs have been performed. The overall percentage of CT acceptance was 86% (701 out of 817 invited persons) and that of MRI was 93% (446 out of 480 invited persons). Since neuroimaging studies have been performed over different study years, some of the invited individuals who initially refused neuroimaging studies, latter accepted them after receiving positive feedback from other members of the community who had neuroimaging done. The main reason for definitively refusing these exams was time constraints, while others reported fear of traveling to a large urban center (including the risk of an accident on the highway). To reduce the rate of refusal, we arranged for relatives or friends to travel together in the same day. Neuroimaging exams were generally well tolerated, with only 16 (2%) persons presenting motion artifacts precluding interpretation of CTs and eight (2%) having claustrophobia during MRI.

#### 3.2.4. Sonographic Studies

The acceptance rate of these exams has been satisfactory, with people declining one or more of these procedures mainly due to time constraints or lack of interest. Ankle-brachial index determinations were performed in 382 (80%) out of 475 invited participants, carotid ultrasounds in 594 (86%) out of 691 invited participants, abdominal ultrasounds in 277 (77%) of 358 invited participants, and transcranial Doppler examinations in 73 (81%) out of 90 invited participants.

#### 3.2.5. Neurophysiological Studies

Electroencephalograms were performed in 163 (91%) out of 180 invited participants, and a single-night diagnostic polysomnography was performed in 144 (84%) out of 172 invited participants. Persons refusing the electroencephalogram reported time constraints or lack of interest (mainly in those who were invited as healthy controls), and those who refused polysomnograms mostly reported that they did not want to sleep away from their homes (or women were not allowed by their husbands).

#### 3.2.6. Cardiac Physiological Tests

These included ambulatory 24-hour Holter monitoring and measurements of aortic arterial stiffness (the latter performed at our center). Holter monitoring was performed in 273 (87%) out of 315 invited participants and measurements of aortic arterial stiffness in 300 (90%) out of 330 invited participants. The main reasons for not performing the Holter monitoring was the fact that individuals had to carry the device 24 hours, and others reported a lack of interest. Those who refused measurements of aortic arterial stiffness reported a lack of interest or time constraints.

#### 3.2.7. Community Lectures/Counseling

As previously noted, three of these programs were conducted (2013, 2015, and 2017). For the first program, 228 (70%) out of 327 invited persons attended, and for the second, 208 (60%) out of 349 persons attended. Most persons who did not attend reported time constraints, while others mentioned a lack of interest. For the third program, however, the attendance increased to 92% (312 out of 338 invited). The most likely reason for this significant increase in attendance was the simultaneous offering of visual acuity determinations and the distribution of prescription reading glasses.

## 4. Discussion

Selection of the village is a fundamental step for successfully conducting a population-based cohort study in a remote rural setting. First, the village has to be representative of the region in terms of population structure, social development, and geographical environment. Second, homogeneous race/ethnic and cultural backgrounds and socioeconomic status of participants reduce the risk of hidden confounders. Third, it is essential that participants are readily available for interviews or diagnostic tests. Fourth, and most important, residents of the selected village must have a low migration rate; otherwise, marked oscillations in population dynamics with a high number of people moving in and out of the village will result in missing data and lack of power for long-term follow-up analyses. Atahualpa meets all these requirements, making it an optimal setting for conducting longitudinal cohort studies [12].

Human factors are also of critical importance to the success of the study. In rural settings, some conditions and diseases are often perceived to be the result of supernatural forces and thus may constitute a stigma that can interfere with educational, work, and social opportunities, or even marriage. Therefore, it is less likely that people with these conditions will readily reveal their symptoms to unacquainted field personnel. In addition, these individuals might refuse diagnostic tests that they believe would disclose their disease or condition. Besides, producing marked heterogeneity in the reported prevalence of some conditions across studies, such denial reduces the chance of participation and, thus, the opportunity to provide appropriate counseling. One of these conditions is epilepsy, which—in people living in rural areas of LMIC—is highly stigmatized. A clear example of denial of disease due to lack of confidence was reported by our group in a survey conducted to estimate the prevalence of epilepsy. Identified persons with epilepsy initially denied their condition to unacquainted field personnel conducting a survey (using the same protocol and instruments) 12 years before, and later, they disclose their condition when familiar personnel conducted the interview [23]. These findings were corroborated in a population-based survey in rural China, where the prevalence of epilepsy increased significantly when the results of a door-to-door survey were complemented with information provided by community leaders and local healers [24]. The same might occur with other conditions, such as cognitive decline or psychological distress, which are often associated with mental illness in these settings. In the Atahualpa Project, appropriate acculturation, meticulous selection of field personnel, and repeated visits to the enrolled population increased people's acceptance of the interviewers and helped to facilitate a high degree of participation.

The high acceptance rate with few refusals may also have been the result of a need for medical attention in remote settings where public health services are inefficient or not available. This may well have produced a higher rate of initial acceptance among villagers. Fatigue to study participation also needs to be considered, particularly if no medications are offered to individuals presenting with symptomatic illness. However, our results do not suggest fatigue of participants since similar acceptance of diverse diagnostic tests have been observed over the course of the study, and most people definitively declined consent only after one or two years following enrollment. Moreover, increasing age and disability, often associated with refusal in other cohort studies [1–3], had no impact on declining consent in the Atahualpa Project, suggesting that, in this rural setting, sick people and the elderly tend to participate in the study, perhaps in order to receive counseling. In contrast, the risk of definitive refusal was higher among relatives or neighbors of a given participant declining consent (Figure 2).

The exception was the attendance at community talks, which revealed a 10% (70% versus 60%) decrease from the first to the second program. However, a simple measure such as providing participants with prescription glasses increased the attendance to 92% during the third program. Creativity is essential to maintain adherence to repetitive procedures (obviously excluding monetary compensation or other kind of incentives). This has also been done with success in other studies attempting to increase participants' retention, where barriers related to increasing age and disability were associated with declining consent [3]. In this view, community integration has been fundamental to maintain compliance to certain time-consuming diagnostic tests such as transportation to Guayaquil for neuroimaging studies. As previously mentioned, some participants who initially refused these exams later consented when their relatives or neighbors encouraged them to accept after sharing their own positive experiences and when we arranged for transportation of their relatives and friends in the same vehicle.

Long-term and appropriate study funding, as well as a lack of commercial conflicts of interest and no interference by the sponsor with decisions taken by the executive board of a given project, also serve to insure the successful realization of a population-based cohort study. In the case of the Atahualpa Project, the sponsor is an academic institution (Universidad Espiritu Santo, Ecuador) without any commercial interest.

A potential limitation of this study is a lack of comparison with other similarly designed studies in rural areas of Latin American countries, which—as previously noticed—do not describe in detail their retention rates and the reasons for declining the long-term follow-up [6, 10]. Further collaborative studies are needed to confirm if our findings may be generalizable to other populations in the region.

## 5. Conclusions

As this study shows, retention in the Atahualpa Project cohort is high, with only 6.3% of definitive refusals after five years of follow-up. This high level of retention is due to several factors including the adequate selection of the village, the detailed planning of procedures, the assurance of sponsorship, and the selection of dedicated field personnel for approaching participants and who satisfactorily explain the main aims of the study and the reasons to participate. The risk of refusal to participate is higher among relatives or neighbors of a participant who declined the consent or among women who are influenced by their declining husbands.

## Figures and Tables

**Table 1:** Characteristics of participants who declined the consent versus those who continued in the cohort.

	Declined consent (*n* = 54)	Continued in the cohort (*n* = 809)	*p* value
Mean age, years ± SD	62.9 ± 12.2	60.4 ± 13.4	0.182
Women, *n* (%)	26 (48.1)	442 (54.8)	0.892
Primary school education, *n* (%)	34 (63)	471 (58.2)	0.469
Stroke or severely disabled, *n* (%)	1 (1.9)	48 (5.9)	0.357

**Table 2:** Procedures and diagnostic tests performed in the population enrolled in the Atahualpa Project cohort and main reasons for refusal.

Diagnostic test	Number (characteristics) of invited individuals	Number (%) of acceptance	Main reasons for refusal
Blood test (year 2013)	616 (aged ≥40 years)	517 (84)	Fear of the needle. Time constraints
Blood test (year 2014)	267 (aged ≥60 years)	220 (82)	
Blood test (year 2015)	351 (aged ≥60 years)	259 (74)	
Blood test (year 2016)	351 (aged ≥60 years)	319 (90)	
Computed tomography^*∗*^	817 (aged ≥40 years)	701 (86)	Time constraints Fear of travel
Magnetic resonance^*∗*^	480 (aged ≥60 years)^§^	446 (93)	
Ankle-brachial index^*∗*^	475 (aged ≥60 years)^§^	382 (80)	Time constraints Lack of interest
Carotid ultrasounds^*∗*^	691 (aged ≥40 years)	594 (86)	
Abdominal ultrasounds	358 (aged ≥40 years)	277 (77)	
Transcranial Doppler	90 (aged ≥60 years)^‡^	73 (81)	
Electroencephalograms^*∗*^	180 (aged ≥40 years)^‡^	163 (91)	
Polysomnography^*∗*^	172 (aged ≥40 years)^‡^	144 (84)	Don't want to sleep out
24-hour Holter monitoring	315 (aged ≥60 years)	273 (87)	Don't want to carry the device for 24 hours
Aortic carotid stiffness	330 (aged ≥60 years)	300 (90)	Time constraints Lack of interest
Community lectures (2013)	327 (aged ≥60 years)	228 (70)	Time constraints Lack of interest
Community lectures (2015)	349 (aged ≥60 years)	208 (60)	
Community lectures (2017)	338 (aged ≥60 years)^¥^	312 (92)	

^*∗*^Exams performed over different study years. ^§^Invitations also extended to certain individuals aged 40–59 years presenting with specific disorders of interest. ^‡^Invitations extended to certain individuals according to study design (not population based). ^¥^Visual acuity determinations and prescription glasses offered to attendants.

## Data Availability

Access to data is restricted as per the policy of our Institution (Universidad Espiritu Santo, Ecuador).
